# Helping Mothers Survive Bleeding After Birth: retention of knowledge, skills, and confidence nine months after obstetric simulation-based training

**DOI:** 10.1186/s12884-015-0612-2

**Published:** 2015-08-25

**Authors:** Ellen Nelissen, Hege Ersdal, Estomih Mduma, Bjørg Evjen-Olsen, Jacqueline Broerse, Jos van Roosmalen, Jelle Stekelenburg

**Affiliations:** Haydom Lutheran Hospital, PO Box 9000 Haydom, Manyara, Tanzania; North Bristol Trust, Southmead Hospital, Bristol, BS10 5NB United Kingdom; Stavanger Acute Medicine Foundation for Education and Research (SAFER), and Department of Anaesthesiology and Intensive Care, Stavanger University Hospital, POB 8100, 4068 Stavanger, Norway; Centre for International Health, University of Bergen, Årstadveien 21, N-5009 Bergen, Norway; Department of Obstetrics and Gynaecology, Sørlandet Hospital, Engvald Hansens vei 6, 4400, Flekkefjord, Norway; Athena Institute, Faculty of Earth and Life Sciences, VU University Amsterdam, de Boelelaan 1085, 1081 HV Amsterdam, The Netherlands; Department of Obstetrics, Leiden University Medical Centre, Albinusdreef 2, 2333 ZA Leiden, The Netherlands; Department of Obstetrics & Gynaecology, Leeuwarden Medical Centre, Henri Dunantweg 2, 8934 AD Leeuwarden, The Netherlands

## Abstract

**Background:**

It is important to know the decay of knowledge, skills, and confidence over time to provide evidence-based guidance on timing of follow-up training. Studies addressing retention of simulation-based education reveal mixed results. The aim of this study was to measure the level of knowledge, skills, and confidence before, immediately after, and nine months after simulation-based training in obstetric care in order to understand the impact of training on these components.

**Methods:**

An educational intervention study was carried out in 2012 in a rural referral hospital in Northern Tanzania. Eighty-nine healthcare workers of different cadres were trained in “Helping Mothers Survive Bleeding After Birth”, which addresses basic delivery skills including active management of third stage of labour and management of postpartum haemorrhage (PPH). Knowledge, skills, and confidence were tested before, immediately after, and nine months after training amongst 38 healthcare workers. Knowledge was tested by completing a written 26-item multiple-choice questionnaire. Skills were tested in two simulated scenarios “basic delivery” and “management of PPH”. Confidence in active management of third stage of labour, management of PPH, determination of completeness of the placenta, bimanual uterine compression, and accessing advanced care was self-assessed using a written 5-item questionnaire.

**Results:**

Mean knowledge scores increased immediately after training from 70 % to 77 %, but decreased close to pre-training levels (72 %) at nine-month follow-up (p = 0.386) (all p-levels are compared to pre-training). The mean score in basic delivery skills increased after training from 43 % to 51 %, and was 49 % after nine months (p = 0.165). Mean scores of management of PPH increased from 39 % to 51 % and were sustained at 50 % at nine months (p = 0.003). Bimanual uterine compression skills increased from 19 % before, to 43 % immediately after, to 48 % nine months after training (p = 0.000). Confidence increased immediately after training, and was largely retained at nine-month follow-up.

**Conclusions:**

Training resulted in an immediate increase in knowledge, skills, and confidence. While knowledge and simulated basic delivery skills decayed after nine months, confidence and simulated obstetric emergency skills were largely retained. These findings indicate a need for continuation of training. Future research should focus on the frequency and dosage of follow-up training.

## Background

Helping Mothers Survive Bleeding After Birth is a new training package for healthcare workers in settings with a high burden of maternal mortality, developed by Jhpiego and Laerdal Global Health. This training programme was created in order to address the lack of practical simulation-based training for frontline healthcare workers in low-resource settings. It uses simulation-based education to train healthcare workers of different cadres in basic delivery skills (including active management of third stage of labour) and management of postpartum haemorrhage (PPH) [1, 2]. Helping Mothers Survive Bleeding After Birth was introduced in a rural referral hospital in Northern Tanzania in March 2012 as part of a pilot study to evaluate and further improve the training programme before global roll out. The pilot study showed that knowledge, skills, and confidence of participants significantly increased immediately after training [3]. This is consistent with systematic reviews showing that simulation-based education results in an immediate and significant increase in knowledge and skills of participants [4, 5].

In order to provide evidence-based guidance on timing of follow-up training it is important to know the decay of knowledge, skills, and confidence over time. Previous studies measuring retention of knowledge and skills after simulation-based education are heterogeneous and reveal mixed results [6, 4, 7–11]. Two systematic reviews regarding retention of resuscitation training show a tendency towards decay of knowledge and skills that may start three months after training, with simulated skills performance deteriorating faster than knowledge [4, 6]. One study evaluating obstetric emergency training showed that decay of knowledge and skills started as early as two months after training [7], however most studies did not show a deterioration of knowledge and skills at six to fifteen months after training [11, 10, 9, 8].

The aim of this study was to measure the level of knowledge, skills, and confidence before, after, and nine months after Helping Mothers Survive Bleeding After Birth simulation-based training to understand the impact of training on these components.

## Methods

An educational intervention study with pre-, post-, and nine-month follow-up assessments was performed from March to December 2012. The Helping Mothers Survive Bleeding After Birth simulation-based training programme was introduced in a rural referral hospital in Northern Tanzania in March 2012. A cross-sectional study that took place in this hospital from November 2009 until November 2011 showed that the maternal mortality ratio was 350 maternal deaths per 100,000 live births (95 % confidence interval: 243–488) [12]. PPH accounted for 27 % of all maternal morbidity and mortality, and the case fatality rate of PPH was as high as 9 % [12]. During the time of this study, the hospital had 420 beds and provided free reproductive services and comprehensive emergency obstetric care. The annual number of births in this period was approximately 4,700 [13].

Helping Mothers Survive Bleeding After Birth uses a train-the-trainer model in which training is cascaded down from master trainers to local facilitators to learners [14]. In two sessions, four master trainers trained eight local facilitators in a one-to-one ratio. Training of local facilitators lasted a full day and consisted of a half-day theory and a half-day skills and scenario teaching regarding basic delivery skills including active management of third stage of labour and management of PPH. Subsequently, these eight facilitators trained 89 local learners in half-day sessions under supervision of master trainers. The number of learners per facilitator ranged from three to six. Clinicians, nurse-midwives, medical attendants (nurse aides without formal medical education), ambulance drivers (without formal medical education), and other staff involved in maternity care (including nurse-midwives from the intensive care unit and operating theatre), were selected by the hospital management to attend training. Due to logistical reasons, only participants working on labour ward, ambulance drivers, and facilitators were enrolled for testing, thus rendering 38 out of the original 89 learners eligible for analysis. Checking competency (or validation) of local facilitators by means of knowledge and skills testing was done after teaching learners. Further details of the training are described elsewhere [3].

The study design was based on the four levels of Kirkpatrick’s model for evaluation of training programmes [15]. In this paper, we report on Kirkpatrick level 2 (learning), for which we have measured changes in knowledge, skills, and confidence due to training. The assessment tools and their validation have been described in detail previously [3]. In brief, knowledge, skills, and confidence were tested on three occasions; immediately before training, immediately after training, and nine months after training. Knowledge about basic delivery skills, active management of third stage of labour, and management of PPH was tested using a written 26-item multiple-choice questionnaire. The criterion-referenced pass score was ≥ 70 % correct answers. The test was developed and assessed for face, content, and construct validity by Jhpiego, of which the details are described elsewhere [3]. Skills performance was assessed in two simulated scenarios using a low-cost, low-tech birthing simulator (MamaNatalie, Laerdal Global Health): “basic delivery” and “management of PPH”. A checklist for the assessment of skills performance was developed and validated by the authors [3]. To pass the test, five essential items for basic delivery, and eight essential items for management of PPH were identified that needed to be performed. Each participant’s skills test was videotaped and subsequently assessed by two independent assessors, who were blinded for the time of testing. Confidence of participants to perform active management of third stage of labour, manage PPH, determine completeness of the placenta, perform bimanual uterine compression, and access advanced care was self-assessed using a questionnaire. Five answers were possible, ranging from 1 = I cannot perform this skill to 5 = extremely confident. At the nine-month assessment all facilitators and learners were interviewed about the number of deliveries performed since initial training, as well as the number of bimanual uterine compressions performed, the number of times MamaNatalie was used for practise, and the participation in any other practise or training regarding basic delivery and management of PPH. All assessment materials were available in two languages, English and Kiswahili (local language).

### Statistical analysis

Data was double entered in EpiData (The EpiData Association, Odense, Denmark), and analysed using IBM SPSS Statistics, version 20 (IBM, Armonk, NY, USA). Descriptive statistics were calculated for participant characteristics, exposure to clinical work and training during the follow-up time, knowledge, skills, and confidence. Results are reported as number (n), percentage (%), mean, standard deviation (SD), and range. Statistical analyses of the changes from pre-training assessment to nine-month follow-up and from post-training to nine-month follow-up included McNemar’s test for categorical values, and paired samples *t*-test for continuous values.

### Ethical approval and informed consent

Ethical approval was obtained from the Tanzanian National Institute for Medical Research (reference NIMR/HQ/R.8a/Vol.IX/1247), the Tanzania Commission for Science and Technology (reference 2013-41-ER-2011-201), and from the VU University Medical Centre, the Netherlands (reference 2011/389). Permission to conduct the study was obtained from the hospital management. Written informed consent was obtained from each participant before entering the study.

## Results

**Fig. 1:**
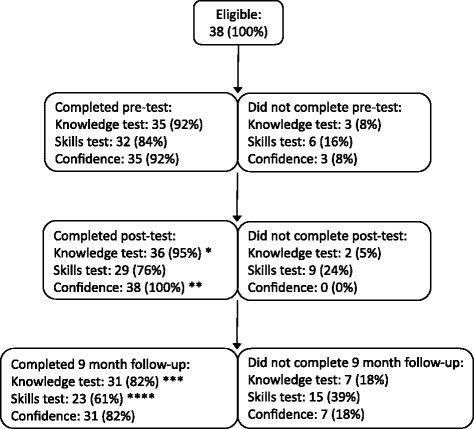
Flow diagram of participants. *Three participants did not complete the knowledge test pre-intervention, but did complete the knowledge test post-intervention and at 9-month follow-up **Three participants did not complete the confidence questionnaire pre-intervention, but did complete the confidence questionnaire post-intervention and at 9-month follow-up ***One participant did not complete the knowledge test post-intervention, but did complete the knowledge test pre-intervention and at 9-month follow-up ****One participant did not complete the skills test post-intervention, but did complete the skills test pre-intervention and at 9-month follow-up

Thirty-eight participants completed the training and were eligible for follow-up (see Fig. 1). Of the 38 participants, 35 (92 %) completed the knowledge test, 32 (84 %) completed the skills test, and 35 (92 %) completed the confidence questionnaire before training. Three participants, who did not complete the knowledge test and confidence questionnaire before intervention, did complete these after intervention and at nine-month follow-up. Immediately after training, 36 (95 %) participants completed the knowledge test, 29 (76 %) completed the skills test, and 38 (100 %) completed the confidence questionnaire. Follow-up of participants at nine months was available for 31 (82 %) participants who completed the knowledge test, 23 (61 %) who completed the skills test, and 31 (82 %) who completed the confidence questionnaire.

The group of participants (n = 38) consisted of six ambulance drivers (16 %), 13 medical attendants (34 %), 14 nurse-midwives (37 %), and five clinicians (13 %) (Table 1). Seventy-four percent were active birth attendants at the time of training. On average, participants assisted 118 births (range 0–840) and performed on average 1.3 bimanual uterine compressions (range 0–10). In nine months, one person used the birthing simulator for practise three times, and one person participated in a training organised by the Tanzanian Ministry of Health regarding management of obstetric emergencies.

**Table 1:** Learner characteristics and clinical activity during 9-month follow-up (n = 38)

Qualification	Ambulance driver, n (%)	6 (16)
	Medical attendant, n (%)	13 (34)
	Nurse-Midwife, n (%)	14 (37)
	Clinician, n (%)	5 (13)
Active birth attendant at time of training, n (%)		28 (74)
Conducted deliveries, mean (range)		118 (0–840)
Conducted bimanual uterine compression, mean (range)		1.3 (0–10)
Times practised with MamaNatalie, mean (range)		0.1 (0–3)
Other practise/training, mean (range)		0.03 (0–1)

Twenty-eight participants completed the knowledge test both before training and at nine-month follow-up. Thirty participants completed the knowledge test both immediately after training and at nine-month follow-up. Knowledge increased immediately after training (from 70 % to 77 %), but was not retained at nine-month follow-up and decreased to pre-training level (72 %; p = 0.386) (Table 2). The results were further analysed for personnel with medical training (clinicians and nurses) and without medical training (medical attendants and ambulance drivers). A similar trend of knowledge decay was seen at nine months. Personnel with medical education achieved higher mean scores and therefore higher pass rates.

**Table 2:** Retention of knowledge among participants

		Pre training	Post training	9 months after training	pre vs. 9-month	post vs. 9-month
					p-value	p-value
All participants						
	Mean score % (SD)	70 (12)	77 (12)	72 (14)	0.386	0.044
	Pass rate n (%)	16 (46)	26 (72)	15 (48)	1.0	0.070
Medically educated personnel						
	Mean score % (SD)	79 (8)	86 (8)	83 (11)	0.646	0.220
	Pass rate n (%)	15 (88)	17 (94)	12 (92)	*na*	1.0
Not medically educated personnel						
	Mean score % (SD)	62 (8)	69 (11)	63 (9)	0.477	0.119
	Pass rate n (%)	1 (6)	9 (50)	3 (17)	0.625	0.070

*na* not available, p-value could not be calculated due to 100 % fail or 100 % pass rate

Twenty-three participants completed the skills test both before training and at nine-month follow-up. Twenty-two participants completed the skills test both immediately after training and at nine-month follow-up. Table 3 shows that participants significantly retained skills in the simulated scenario of management of PPH (mean score of 50 %) and bimanual uterine compression (mean score of 48 %) at nine-month follow-up, compared to pre-training level (mean score of 39 % for management of PPH [p = 0.003], and 19 % for bimanual uterine compression [p = 0.000]). Skills in the simulated scenario of basic delivery increased after training (from 43 % before training to 51 % immediately after training), but this increase was not sustained at nine-month follow-up (49 % [p = 0.165]). This analysis was further specified for personnel with and without formal medical education. It showed that personnel with medical education did not manage to retain basic delivery skills that were gained immediately after training; the mean performance of clinicians and nurse-midwives was 57 % before training, 61 % immediately after training and 55 % nine months after training (p = 0.723) (Table 3). Personnel without medical education retained all skills at nine months compared to before training; the mean performance of medical attendants and ambulance drivers was 29 % before training, 43 % immediately after training and 44 % nine months after training (p = 0.026). In general, personnel with medical training achieved higher mean scores compared to personnel without medical training. However, medical attendants and ambulance drivers showed a greater improvement in skills gained and retained compared to baseline. Overall, pass rates of participants were low.

**Table 3:** Retention of skills among participants

		Pre training	Post training	9 months after training	pre vs. 9-month	post vs. 9-month
					p-value	p-value
All participants						
Basic delivery						
	Mean score % (SD)	43 (22)	51 (19)	49 (19)	0.165	0.062
	Pass rate n (%)	0 (0)	1 (3)	1 (4)	*na*	*na*
Management of PPH						
	Mean score % (SD)	39 (27)	51 (21)	50 (24)	0.003	0.724
	Pass rate n (%)	2 (6)	1 (3)	2 (9)	1.0	1.0
Bimanual uterine compression						
	Mean score % (SD)	19 (20)	43 (25)	48 (25)	0.000	0.178
Medically educated personnel						
Basic delivery						
	Mean score % (SD)	57 (16)	61 (13)	55 (16)	0.723	0.221
	Pass rate n (%)	0(0)	1 (8)	0(0)	*na*	*na*
Management of PPH						
	Mean score % (SD)	61 (16)	67 (9)	71 (14)	0.154	0.281
	Pass rate n (%)	2 (13)	1 (8)	2 (20)	1.0	1.0
Bimanual uterine compression						
	Mean score % (SD)	33 (18)	53 (16)	55 (21)	0.017	0.212
Not medically educated personnel						
Basic delivery						
	Mean score % (SD)	29 (19)	43 (21)	44 (21)	0.026	0.188
	Pass rate n (%)	0 (0)	0 (0)	1 (8)	na	na
Management of PPH						
	Mean score % (SD)	17 (15)	39 (19)	33 (16)	0.005	0.148
	Pass rate n (%)	0 (0)	0 (0)	0 (0)	na	na
Bimanual uterine compression						
	Mean score % (SD)	4 (8)	35 (28)	42 (28)	0.001	0.445

*na* not available, p-value could not be calculated due to 100 % fail or 100 % pass rate

**Fig. 2:**
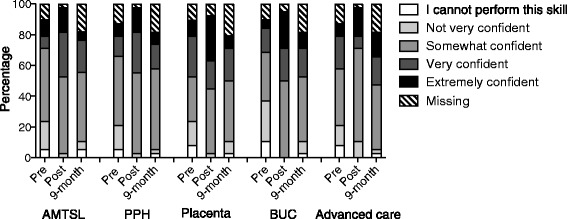
Distribution of confidence of participants pre-training, immediately post training and at 9-month follow-up. AMTSL: Active Management of Third Stage of Labour, PPH: Postpartum Haemorrhage, Placenta: determine completeness of placenta, BUC: Bimanual Uterine Compression, Advanced care: Access advanced care

Figure 2 shows the distribution of confidence among 38 participants before training, after training, and at nine-month follow-up. Confidence in managing PPH, bimanual uterine compression, and accessing advanced care remained significantly elevated at nine-month follow-up, compared to pre-training levels (p = 0.029, p = 0.008, and p = 0.029 respectively). Confidence in performing active management of third stage of labour and determining the completeness of the placenta was not retained at nine-month and returned to pre-training levels (p = 0.282 and p = 0.294 respectively).

## Discussion

Our findings show that knowledge increased immediately after training, but decreased to pre-training levels when re-assessed nine months later. Simulated performance of emergency skills in the management of postpartum haemorrhage were retained after nine months. However, simulated performance of basic delivery skills by medically educated personnel decayed. In a similar fashion, confidence increased immediately after training, and was retained in three out of five areas (management of PPH, bimanual uterine compression, and accessing advanced care) at nine-month follow-up.

After an initial increase, knowledge in general, and basic delivery skills of medically trained personnel were not retained at nine months follow-up. We speculate that everyday tasks (e.g. basic delivery) are engrained into someone’s behaviour and therefore may be more difficult to change than less common tasks (e.g. management of PPH, bimanual uterine compression) [16, 17]. For example, bimanual uterine compression was a new skill for most healthcare workers, therefore not influenced by former experience, and was better retained after training. This finding indicates a clear need for continuation of training in order to maintain knowledge and routine obstetric skills of healthcare workers.

Simulated obstetric emergency skills such as management of PPH and bimanual uterine compression were largely retained over nine months, despite a decrease in knowledge scores. Part of this may be explained by the practical focus of the Helping Mothers Survive Bleeding After Birth training programme. The mannequin (MamaNatalie, Laerdal Global Health) used in this training programme has very realistic features that aid the training of emergency skills, such as the ability to bleed up to 1.5 litres of blood, a uterus that can contract and relax, and the possibility to perform bimanual uterine compression.

Furthermore, the number of steps required in our scoring could have influenced retention. The more steps that need to be remembered, the easier it is to forget. In general, people can remember up to nine steps [18]. In our study, scoring for basic delivery consisted of 16 steps, management of PPH consisted of 12 steps, and bimanual uterine compression consisted of six steps. The lesson that can be learned here is that algorithms, such as the action plan used in the Helping Mothers Survive Bleeding After Birth training programme [2], should be as brief as possible, focussing only on the most important actions.

In this study we have measured simulated performance. It is not known how simulated obstetric skills translate into real practise. A study by Ersdal et al. showed that improved simulated performance in neonatal resuscitation skills did not transfer into clinical practise [19]. Therefore, it is of importance to include measurement of clinical practise and patient outcome (Kirkpatrick level 3 and 4) in the evaluation of this training programme.

Our study was part of a pilot study. Results of this study have been used to further improve Helping Mothers Survive Bleeding After Birth. Jhpiego has incorporated suggested improvements (full day training, translation of teaching materials into local language, and validation of facilitators) into the latest version of the training programme [2]. A follow-up study by Evans et al. is promising and shows a significant increase in knowledge and confidence in three different settings (India, Malawi, Zanzibar) [20]. Passing scores of post training skills tests ranged from 83 % to 89 % [20].

Future research should focus on the most effective way to continue training and determinants of effective implementation of training programmes. Questions that need answering are how often training should take place (once a day, once a week, once a month, every six months, or every year), and how it should be dosed (focussing on one item in a scenario such as bimanual uterine compression, or a full scenario such as PPH due to uterine atony).

A limitation of this study is the before and after design; there was no control group and no randomisation. Factors other than time may have influenced the effect of training on retention of knowledge, skills, and confidence. Therefore, potential sources of bias were measured before and after training. However, there was no additional training, no introduction of other guidelines, and no change of leadership. There was turnover of staff, but no increase of staff during the study period. As we tested the same people before and after training, this should not have influenced the results. Secondly, the study was carried out in one hospital setting, which makes external validation difficult. The fact that the sample size was small and the group was heterogeneous (with and without medical training) may further affect generalisability. We plan to remedy these limitations by evaluating all four levels of the Kirkpatrick model for the same training intervention. When results of each level reinforce each other, it is more likely that a potentially improved performance of healthcare workers is the effect of training and not just by chance alone. Another consideration for the interpretation of this study is that on all three testing occasions similar knowledge questionnaires and simulated scenarios were used. Participants may have learned from retaking the same test, and this may have resulted in a potentially larger learning effect. As this reflects the training as it is currently given, and the effect applies to all participants, we considered this limitation as minor.

## Conclusions

In conclusion, a half-day obstetric simulation-based training course resulted in an immediate increase in knowledge, simulated skills, and confidence. While knowledge and simulated basic delivery skills of medically trained staff decayed after nine months, basic delivery skills, emergency obstetric skills, and related confidence of staff without medical training were retained. These findings indicate a clear need for continuation of training. Future research should focus on the frequency and dosage of follow-up training.

## Abbreviation

PPH: Postpartum haemorrhage
